# Irrigation depth far exceeds water uptake depth in an oasis cropland in the middle reaches of Heihe River Basin

**DOI:** 10.1038/srep15206

**Published:** 2015-10-14

**Authors:** Bin Yang, Xuefa Wen, Xiaomin Sun

**Affiliations:** 1 Key Laboratory of Ecosystem Network Observation and Modeling, Institute of Geographic Sciences and Natural Resources Research, Chinese Academy of Sciences, Beijing 100101, China; 2 University of Chinese Academy of Sciences, Beijing 100049, China

## Abstract

Agricultural irrigation in the middle reaches of the Heihe River Basin consumes approximately 80% of the total river water. Whether the irrigation depth matches the water uptake depth of crops is one of the most important factors affecting the efficiency of irrigation water use. Our results indicated that the influence of plastic film on soil water *δ*^18^O was restricted to 0–30 cm soil depth. Based on a Bayesian model (MixSIR), we found that irrigated maize acquired water preferentially from 0–10 cm soil layer, with a median uptake proportion of 87 ± 15%. Additionally, maize utilised a mixture of irrigation and shallow soil water instead of absorbing the irrigation water directly. However, only 24.7 ± 5.5% of irrigation water remained in 0–10 cm soil layer, whereas 29.5 ± 2.8% and 38.4 ± 3.3% of the irrigation water infiltrated into 10–40 cm and 40–80 cm layers. During the 4 irrigation events, approximately 39% of the irrigation and rainwater infiltrated into soil layers below 80 cm. Reducing irrigation amount and developing water-saving irrigation methods will be important strategies for improving the efficiency of irrigation water use in this area.

Due to the scarcity of rainfall, more than 40% of global food production comes from the 20% of agricultural lands that are irrigated^1^. Accordingly, irrigated agriculture is the largest consumer of water resources and utilises 70–90% of the world’s withdrawal of freshwater^2^. In particular, for arid and semiarid areas, irrigation will directly determine development of agrarian production. Over 60% of China’s freshwater is also used for agricultural irrigation^3^. However, the irrigation water use efficiency is only approximately 40%^4^. In these irrigated croplands, the traditional flood irrigation depths frequently exceed 100 cm^5^,^6^,^7^. Therefore, whether the irrigation depth matches the water uptake depth of crops is a primary factor affecting the efficiency of irrigation water use.

The oxygen and hydrogen isotope compositions (*δ*^18^O and *δ*D) of xylem water can indicate the potential depth of plant water sources due to the absence of isotopic fractionation during water uptake by plant roots^8^. Numerous studies have described water uptake patterns in forests^9^,^10^,^11^ and grasslands^12^,^13^,^14^, but little attention has been focused on croplands. Studies have assessed the main water uptake depths for monocultures^15^,^16^,^17^ and the competitive interactions among intercropped plants^18^,^19^,^20^ by directly comparing *δ*^18^O and *δ*D in xylem and soil water. Other studies have identified the proportional contributions of water from each soil depth to crops according to the isotopic mixing models^21^,^22^. Compared to previous mixing models (e.g., IsoSource^23^,^24^), Bayesian models (e.g., MixSIR^25^,^26^, RAPID^27^ and SIAR^28^) have greater statistical power to incorporate uncertainty associated with isotope signatures and prior information^29^. However, we should also note that these models remain a statistical-based tool that only provides a range of feasible solutions and the *“best bet”*.

In an irrigation event or over longer time scales, the deep percolation of irrigation water is mainly quantified based on soil water balance equations, enabling the water use efficiency and proper scheduling of irrigation to be assessed^6^,^7^,^30^. This method is usually time-consuming and labour intensive because it requires data on precipitation, irrigation, evapotranspiration, surface runoff and changes in soil water content. Nevertheless, the direct measurement of the soil water dynamic is often difficult (especially if the number of site-specific measurements is limited) due to the spatial variability of the soil water content during periods following irrigation events. In addition, it is difficult to determine the amounts of irrigation water infiltrating to the different soil layers. Rowland *et al*.^31^ attempted to quantify the irrigation water infiltration through 0–40 cm soil profiles using *δ*D-labelled water in a peanut (*Arachis hypogaea* L.) field. The study demonstrated that 32%, 18% and 8% of irrigation water (8.5 mm) was retained in the 0–10 cm, 10–20 cm and 20–40 cm soil layers, respectively, with the remaining 42% lost to runoff. However, whether the naturally occurring differences in *δ*^18^O and *δ*D in irrigation and soil water could be used to quantify the infiltration of irrigation water has not been reported in the literature.

The artificial oasis cropland in the middle reaches of the Heihe River Basin is the largest maize seed reproduction centre in China. These oases are patchily distributed and surrounded with fixed or semi-fixed sand dunes, in which agricultural production is maintained by irrigation. Irrigation agriculture in this region consumes more than 80% of the water of Heihe River^32^. The maize was typically irrigated 1 time before sowing (mid-to-end of March) and 4 times during the growing season (June to August, at 25–30 day intervals). However, the water use efficiency is very low because the excess water from irrigation is most likely to be wasted by severe evaporation and used to recharge the groundwater^4^,^33^. Under long term cultivation and irrigation, the topography of the cropland is fairly smooth with sandy and loamy soil. The expansion of agriculture in the middle reaches of the Heihe River Basin has also resulted in serious ecological problems, such as vegetation degradation, the decline of the groundwater table and desertification in the lower reaches^34^. Since 2000, a series of institutional water regulations have been in force with the aim of reducing the water used for irrigation in the middle reaches and maintaining the sustainable development of agriculture in the lower reaches. However, we suspected that the maize planted in this area mainly used surface soil water and that the irrigation depth far exceeded that of the root water uptake. The objectives of this study were to (1) investigate the seasonal water uptake depth of maize based on *δ*^18^ O, (2) attempt to utilise *δ*^18^O to quantify the proportion of irrigation water infiltrating to different soil strata after irrigation, and (3) evaluate the deep percolation during the periods of 4 irrigation events based on the soil water balance equation.

## Results

### Effect of film mulching on isotopic profiles of soil water

**Figure 1:**
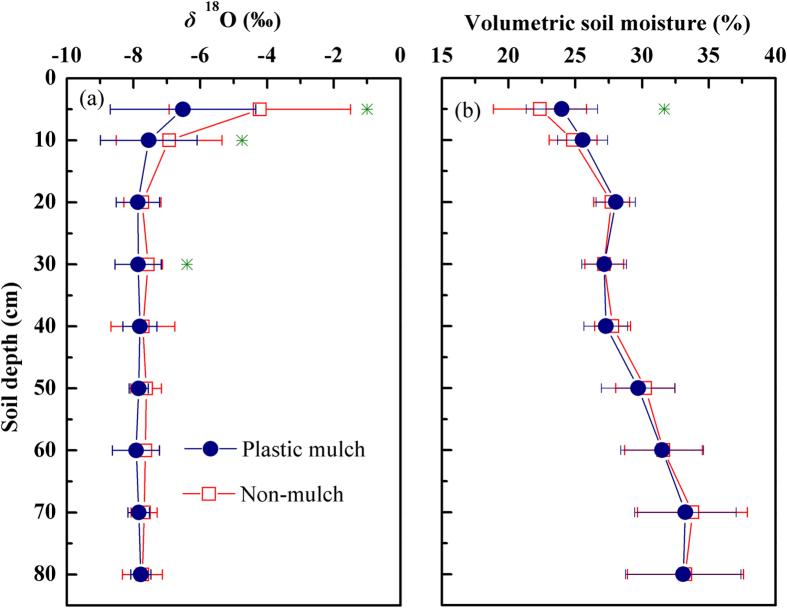
Mean *δ*^18^O (a) and soil water content (SWC) (b) for plastic-mulched and non-mulched soil water in 0–80 cm of a desert artificial oasis cropland in Zhangye. The error bars indicate one standard deviation. * besides a depth interval indicates statistically significant difference (*p* < 0.05).

**Figure 2:**
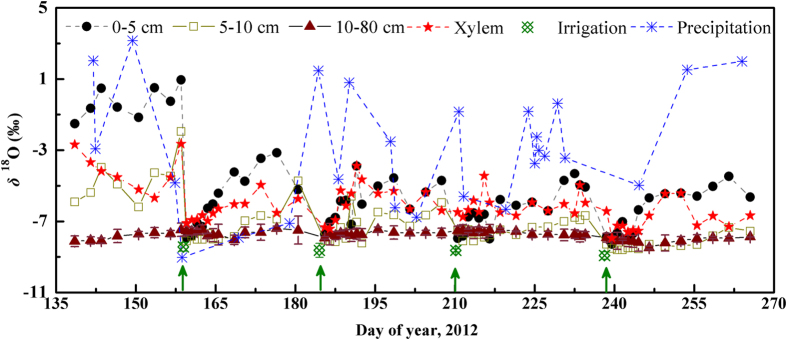
Seasonal variations of *δ*^18^ O in soil water (0–5, 5–10 and 10–80 cm), xylem water, irrigation water and precipitation of a desert artificial oasis cropland in Zhangye. The arrows indicate the dates of irrigation.

Figure 1 presents the mean *δ*^18^O and soil water content (SWC) for plastic-mulched and non-mulched soil water in 0–80 cm during the maize growing season. The *δ*^18^O of soil water is mainly influenced by two independent processes, which are evaporation and mixing with precipitation^35^. Between precipitation events, evaporation from non-mulched soil would decrease SWC of unsaturated zone and result in a progressive enrichment of *δ*^18^O in the residual soil water. The *δ*^18^O of mulched soil, however, was less enriched at 0–30 cm depths (*p* < 0.05), because film mulching weakened the evaporation process (i.e., water retention effect). The water evaporated from mulched soil was transformed to vapour and then condensed into drops on the surfaces of soil and plastic films. This could be confirmed by the results that SWC and soil temperature of the mulched soil in the 0–5 cm depth increased by 1.7 ± 3.1% and 0.7 ± 1.1 °C (*p* < 0.05), respectively. Generally, precipitation for a rainfall event was either infiltrated into the non-mulched soil (increased SWC) or evaporated directly from the mulching surface (contributed to evapotranspiration, *ET*). Although the amount of rainfall directly infiltrated into soil surface was not quantified, *δ*^18^O of the non-mulched soil would be further enriched because precipitation *δ*^18^O was normally higher than that of soil water during the growing season (Fig. 2). The mean *δ*^18^O of mulched and non-mulched soil in 0–80 cm were −7.85 ± 1.26% (ranged from −9.64 to 0.56%) and −7.63 ± 1.82% (−9.17 to 2.67%), respectively.

**Figure 3:**
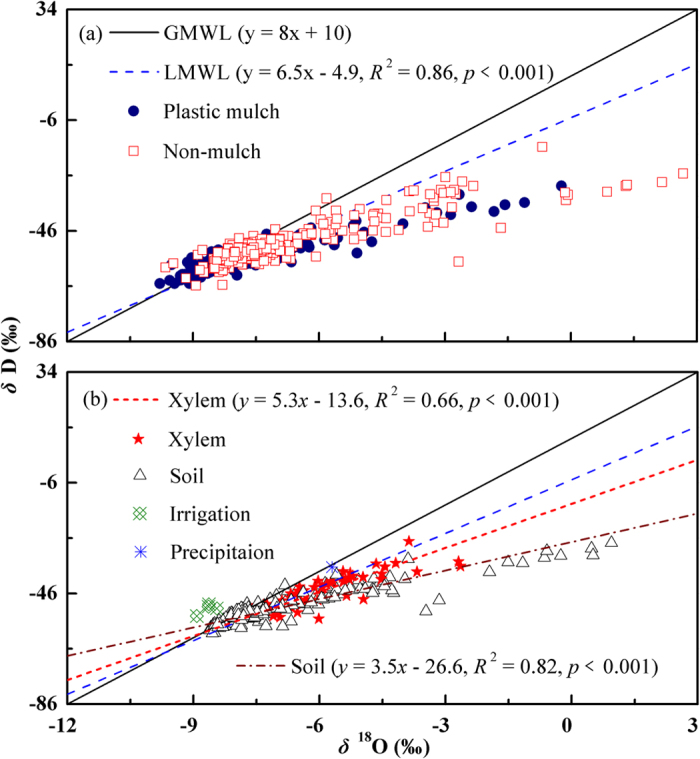
Values of *δ*D as a function of *δ*^18^O for plastic-mulched and non-mulched soil water in 0–80 cm (a), soil water after combination in 0–80 cm (plastic mulch: non-mulch = 5:5), xylem water, irrigation water and average precipitation (weighted by rain amount) (b) of a desert artificial oasis cropland in Zhangye. Data were fitted against the local meteoric water line (LMWL). The global meteoric water line (GMWL) is also presented.

Figure 3a presents the linear dependence of *δ*^18^O on *δ*D for mulched and non-mulched soil in 0–80 cm. The geometric mean regression (GMR) was used in this study because the two variables of the regression equation were random and subjected to errors. The local meteoric water line (LMWL: y = 6.5x–4.9, *R*^2^ = 0.86, *p* < 0.001) fitted based on the precipitation data was similar to that reported in another study in this area (y = 6.8x–4.5)^36^. The slope and intercept of the LMWL were less than those of the global meteoric water line (GMWL: y = 8x + 10), reflecting evaporation in the process of precipitation^21^. A subset of *δ*^18^O and *δ*D in mulched (y = 3.9x–23.9, *R*^2^ = 0.81, *p* < 0.001) and non-mulched (y = 3.9x–24.0, *R*^2^ = 0.78, *p* < 0.001) soil plotted on the LMWL and indicated that soil water in deep layers was not affected by evaporation. The other parts of the *δ*^18^O and *δ*D data plotted to the right of the LMWL reflected the enrichment associated with evaporation in surface soil layers. Furthermore, the *δ*^18^O and *δ*D of the mulched soil was closer to the LWML, which may be the result of the water retention effect of film mulching.

Maize may absorb soil water from both sides of its roots; therefore, the *δ*^18^O of the mulched and non-mulched soil at the same depth must be combined (e.g., according to the film cover rate of the field). The initial film cover rate was approximately 54% (i.e., mulched: non-mulched soil = 7:6). However, the actual cover rate was very close to 50% due to the manual holes excavated for sprouts and the natural shrinkage of the films. Therefore, we first combined the mulched and non-mulched soil isotope data at a ratio of 5:5.

### Seasonal variations in isotopic compositions of water pools

Figure 2 presents the seasonal variations of *δ*^18^O in soil water (0–5 cm, 5–10 cm and 10–80 cm), xylem water, irrigation water and precipitation during the growing season. The soil water *δ*^18^O of the 0–5 cm depth differed markedly from that in 5–10 cm (*p* < 0.001) and in 10–80 cm (*p* ≤ 0.006). There was no significant difference for *δ*^18^O among the depths of 10–80 cm soil stratum (10–20 cm, 20–30 cm, 30–40 cm, 40–50 cm, 50–60 cm, 60–70 cm and 70–80 cm) (*p* > 0.05). Therefore, 0–80 cm soil stratum were divided into three depths (0–5 cm, 5–10 cm and 10–80 cm) to represent the potential water sources for maize. The *δ*^18^O values generally decreased from shallow (0–5 cm) to deep (10–80 cm) soil, except during the days after irrigation. During the growing season, the average soil water *δ*^18^O at 0–5 cm and 5–10 cm was −5.32 ± 2.32% (−8.29 to 0.95%) and −7.13 ± 1.37% (−8.61 to −1.96%), respectively. Soil water *δ*^18^O in 10–80 cm was relatively constant, approaching the value of 7.77 ± 0.23% (−8.49 to −7.40%). The seasonal variability of xylem water *δ*^18^O was similar to that of shallow soil water, reflecting the influence of rainfall and irrigation; the average value was **−**6.10 ± 1.13% (−7.96 to −2.65%). Note that xylem water *δ*^18^O was typically less negative than that of the soil water at 0–5 cm within 7 days after irrigation. Most likely, the maize utilised extremely shallow soil water (<2.5 cm) during this period. The irrigation water *δ*^18^O was distinguished from that of other water pools with a mean value of −8.66 ± 0.20% (−8.92 to −8.44%). Weighted by the rainfall amount, the average precipitation *δ*^18^O was −5.70% (−9.03 to 3.16%) and fluctuated widely during the growing season.

Figure 3b presents the linear dependence of *δ*^18^O on *δ*D for combined soil water (plastic mulch: non-mulch = 5:5), xylem water, irrigation water and average precipitation (weighted by rainfall amount). The slope and intercept of the combined soil water (y = 3.5x–26.6, *R*^2^ = 0.82, *p* < 0.001) were less than those of mulched and non-mulched soil water. Although evaporation could account for the *δ*^18^O and *δ*D of soil water plotting to the right side of LWML, the *δ*^18^O and *δ*D values falling along the line were mainly related to the greatly changed precipitation during the growing season^37^. The *δ*^18^O and *δ*D of xylem water (y = 5.3x–13.6, *R*^2^ = 0.66, *p* < 0.001) plotted between irrigation and soil water, suggesting that they primarily utilised irrigation-recharged soil water. Representing the irrigation water of the field, the *δ*^18^O and *δ*D of the Heihe River water plotted to the left side of the LWML. Because 80–90% of the Heihe River water originates from the upper mountainous area^38^, this finding was also supported by the fact that precipitation (in the form of glacial snowmelt water) decreases with increased altitude or during transport from coastal areas to the inland areas^39^. This depleted feature of irrigation water *δ*^18^O and *δ*D also enables the infiltration of irrigation to be analysed using isotopic methods.

### Seasonal variations in the depth of water uptake by maize

**Figure 4:**
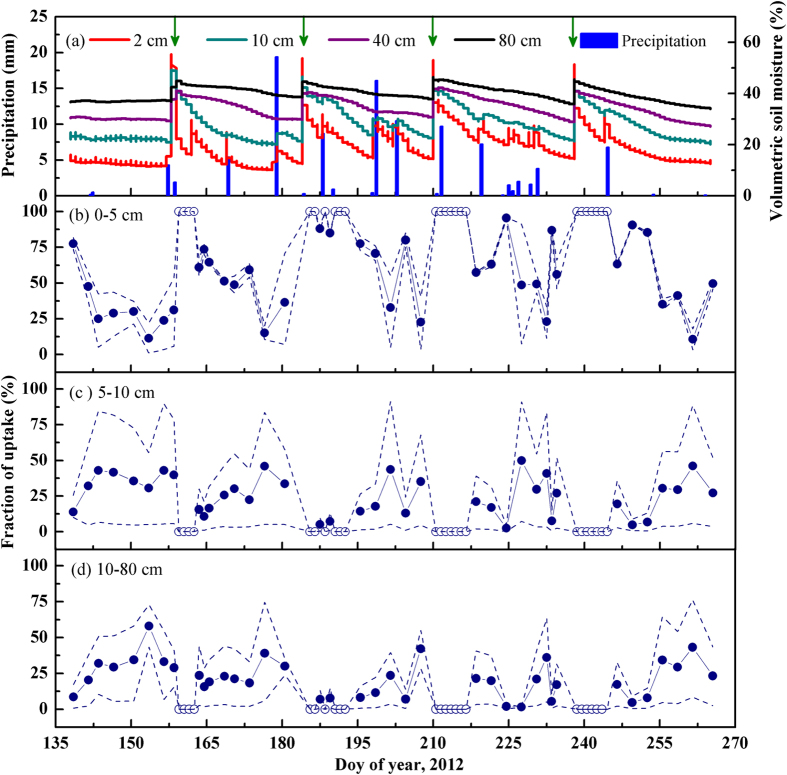
Seasonal variations of soil water content (SWC), rainfall amount (a) and fractions of water uptake from 0–5 (b), 5–10 (c) and 10–80 cm (d) soil depths for maize of a desert artificial oasis cropland in Zhangye. Solid circle and dashed lines represent the medians and percentiles (they are the 5 and 95% confidence percentiles) of MixSIR model solutions. Open circles indicate maize might derive all of its water (~100%) from surface 0–5 cm soil layer after irrigation. The arrows indicate the dates of irrigation.

Figure 4a presents the seasonal variations of the soil water content (SWC) and rainfall amount. During the growing season, SWC of the 0–80 cm depth (2 cm, 10 cm, 40 cm and 80 cm) increased incrementally from surface to deep soil layers. Among these layers, SWC of 2 cm was highly variable due to the influence of precipitation and irrigation. However, soil water recharged by precipitation was confined within 0–10 cm due to the low rainfall during each event. After irrigation, SWC of all layers showed a significant rise and gradually decreased.

The proportional contributions of the 0–5 cm, 5–10 cm and 10–80 cm depths’ soil water to maize were assessed by MixSIR (Fig. 4b–d), which did not consider the spatial heterogeneity of *δ*^18^O in xylem and soil water (standard deviation, SD = 0). When *δ*^18^O of xylem was less negative than that of soil, we assumed that all of the water for maize was from the topsoil at 0–5 cm (see section “Seasonal variations in isotopic compositions of water pools”). The water uptake pattern of maize changed periodicity during the growing season. Before the first irrigation, the contribution of water from 0–5 cm soil layer dropped from a median of 78% (74–83%, this and the following represent the 5 and 95% confidence percentiles) to 31% (6–54%). Although the maximum contribution of water from 10–80 cm depth accounted for 58% (43–73%) during this period, we inferred that the depth of the water source for maize was just over 10 cm due to the relatively shorter roots at the seedling stage. After each irrigation, the contribution of water from 0–5 cm depth decreased from 100% to 36 (7–71%), 23 (4–40%), 56 (46–66%) and 50 (46–53%), respectively. Accordingly, the absorption of water from 5–10 cm increased to 34 (5–58%), 35 (5–68%), 27 (3–52%) and 27 (4–52%), respectively. Meanwhile, water from 10–80 cm increased to 30 (24–35%), 42 (28–55%), 17 (2–32%) and 23 (3–43%), respectively. As the SWC of the 0–10 cm depth increased rapidly after the 5.4 mm (DOY 169), 19.4 mm (DOY 178), 10.5 mm (DOY 202), 7.2 mm (DOY 219), 3.8 mm (DOY 230) and 6.8 mm (DOY 244) rainfall, maize increased the utilisation of the 0–10 cm soil water again (Fig. 4b,c). During the growing season, the median contributions of the 0–5 cm, 5–10 cm and 10–80 cm depths’ soil water were 71 ± 30%, 16 ± 17% and 13 ± 15%, respectively. A root excavation conducted in the late growing season (DOY 240) suggested that 65.5 ± 8.3% of root biomass concentrated in the 0–10 cm soil layer, which was hopeful in backing up the results from the isotopes.

### Infiltration of irrigation water to different soil layers

**Figure 5:**
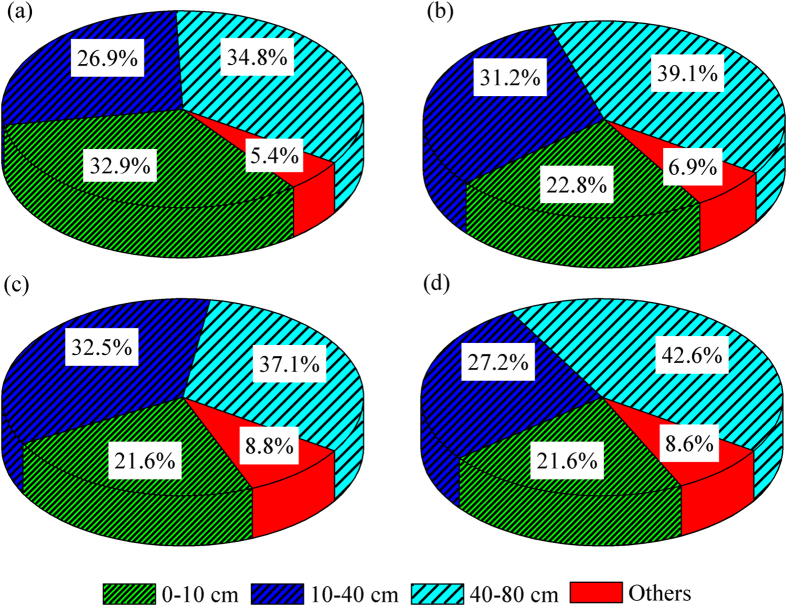
Fates of the 111.6 (a), 141.9 (b), 149.7 (c) and 149.7 mm (d) irrigation water after irrigation of a desert artificial oasis cropland in Zhangye.

Figure 5 presents the fates of the 111.6 mm, 141.9 mm, 149.7 mm and 149.7 mm irrigation water. Rowland *et al*.^31^ found that the labelled irrigation water *δ*D was greatly enriched (i.e., 4300%; the local soil water was close to −30%). Therefore, they could quantify the water movement through the soil layers over a 4 day period. However, we merely analysed the water infiltration after the first day of irrigation, mainly because the difference in *δ*^18^O between the naturally occurring irrigation and soil water was relatively small in this cropland. All of the samples were collected at midday within 13 hours, 24 hours, 9 hours and 10 hours after irrigation. According to Equation (3), 24.7 ± 5.5% (33.4 ± 2.2 mm) of the irrigation water infiltrated into the 0–10 cm soil layer and 29.5 ± 2.8% (40.9 ± 7.9 mm) and 38.4 ± 3.3% (53.4 ± 10.5 mm) of this water reached the 10–40 cm and 40–80 cm soil layers, respectively. In addition, 7.4 ± 1.6% of the irrigation water was lost to evaporation, etc.

If all of the irrigation water (from 4 irrigation of 111.6 mm, 141.9 mm, 149.7 mm and 149.7 mm) infiltrated into the 0–80 cm soil layer after irrigation, we could calculate that, in total, 288.1 mm, 317.9 mm, 283.0 mm and 316.0 mm, respectively, of this water was retained in the 0–80 cm soil profiles, according to Equation (4). The soil water storage in the 0–80 cm layer were 272.7 mm, 295.8 mm, 258.1 mm and 288.5 mm after irrigation, according to the direct SWC measurements. The two methods provided similar results. However, values acquired using the isotopic approach were slightly larger than those of the latter method (after irrigation, soil water storages increased by 14.5 mm, 22.1 mm, 24.9 mm and 27.5 mm). This difference could be attributed to the fact that irrigation water did not completely infiltrate into the 0–80 cm soil layer. Therefore, the results calculated based on the isotopic approach would be larger. Similarly, 176.5 mm, 176.0 mm, 133.3 mm and 166.3 mm of irrigation water was retained in the 0–80 cm soil layer before irrigation according to the isotopic approach, whereas the values acquired by direct measurement were 162.8 mm, 196.4 mm, 187.7 mm and 207.7 mm, respectively, revealing a notable increase in the difference between the two methods (13.7 mm, 20.4 mm, 54.4 mm and 41.4 mm, respectively). This difference was most likely due to the lack of representativeness of the site-specific measurement of the SWC before irrigation. However, after irrigation, the spatial heterogeneity of the SWC was weakened. As a consequence, the results of these two methods were in good agreement.

### Deep percolation after the irrigation events

Table 1 lists the deep percolation of irrigation water and rainfall after the 4 irrigation events. Here, we defined an irrigation event as the period between the start of an irrigation event and the start of the next one. Therefore, the growing season of maize was roughly divided into 4 irrigation events of I (DOY 158-184), II (DOY 184-210), III (DOY 210-238) and IV (DOY 238-265). As mentioned above (see section “Effect of film mulching on isotopic profiles of soil water”), precipitation was either infiltrated into the non-mulched soil or evaporated from the mulching surface. We did not quantify the amounts of precipitation lost to these two processes. However, the total changes in soil water storage (*ΔW*) and evapotranspiration (*ET*) were measured during the study period, which contained the infiltration and evaporation of precipitation, respectively. Although measurement of *ΔW* was always inaccurate (see section “Infiltration of irrigation water to different soil layers”), it would not have much impact on the estimation of the soil water balance due to its relatively small order of magnitude. Based on Equation (5), the deep percolation was 18.7 mm, 81.4 mm, 81.9 mm and 73.0 mm after the 4 irrigation events. Except for the first irrigation event (14%), the percentage of deep percolation accounted for approximately 46% of the irrigation and rainwater. During the periods of the 4 irrigation events, 39% (265 mm) of the irrigation and rainwater was lost to the soil layer below 80 cm. Another study conducted in this area also reported that 22–39% (133–330 mm) of the irrigation and rainwater was lost to deep percolation below 200 cm^7^.

## Discussion

The sensitivity analysis of the median water uptake fractions from different soil layers to the ratios of plastic-mulched to non-mulched soil is shown in Table 2. To the best of our knowledge, no previous study has presented a method for determining the water uptake depth of crops in a plastic-mulched field. In the above analyses, *δ*^18^O of mulched and non-mulched soil at a certain depth was combined together according to the film cover ratio of 5:5. Theoretically, *δ*^18^O of mulched and non-mulched soil water should be combined according to soil water content (SWC). In order to consider the spatial heterogeneity of SWC in the field (see section “Deep percolation after the irrigation events”), however, the ratios of plastic-mulched to non-mulched soil could provide an effective substitute for SWC in the sensitivity analysis. In fact, there was little difference between the outcomes of MixSIR by these two methods (*p* = 0.977). When compared to the results acquired by the combination ratio of 5:5, adopting the ratio of 6:4 and 4:6 would not significantly impact the prediction of water uptake depth (*p* was 0.43 and 0.23, respectively). However, combining the *δ*^18^O of mulched and non-mulched soil by the ratio of 3:7 and 2:8 would significantly influence the predictions (*p* = 0.04 and 0.006, respectively).

The sensitivity analysis of the median water uptake fractions from the different soil layers to model uncertainty is shown in Table 3. In this study, we did not take into account the spatial heterogeneity of *δ*^18^O in xylem and soil water during the sampling. However, the uncertainty associated with isotope signatures might influence the prediction of plant water sources^9^,^25^. Using the standard deviation (SD) of isotope data in the published literature^17^,^20^,^22^, we evaluated the possible influence of the spatial heterogeneity of *δ*^18^O on the water source prediction. When compared to the results acquired by MixSIR_1 model (SD = 0), the use of different SDs (SD = 0.6%, 0.9% and 1.5%, respectively) did not significantly affect the water source partitioning (*p* ≥ 0.112). When the SD of isotope data was not considered, the results acquired by the MixSIR and IsoSource models were almost identical (*p* = 0.92).

It should be noted that MixSIR model would lead to multiple solutions. Moore and Semmens^25^ have validated the performance of the model by developing artificial data. It was difficult to validate our results directly with the data of this study. However, the model predictions could be proved to be reasonable by the fact that these results were consistent with that obtained by directly comparing *δ*^18^O in xylem and soil water (Fig. 2). Furthermore, the measured root distribution in this study was also in accordance with the model predictions.

A few of studies reported that the main water source of maize was restricted to shallow depths for both irrigated^17^,^21^ and non-irrigated maize^40^. The obvious difference of *δ*^18^O in xylem and irrigation water indicated that maize did not directly absorb irrigation water after irrigation, which was also reported in a rice (*Oryza sativa* L.) cropland^22^. In annual crops, extracting water from surface soil might facilitate the absorption of other nutrients (e.g., N, P and K), which were highest in the shallow layers of soil. We also found that maize adjusted quickly to the uptake of shallow soil water in response to irrigation or heavy rainfall. As the SWC of the 0–10 cm layer decreased, the maize utilised more water from the 10–80 cm soil layers. This finding suggested that crops exhibit plasticity with respect to the depth of water uptake, which is quite common for annual and perennial plants in arid and semiarid ecosystems^16^. Nevertheless, other studies also found that maize can tap soil water at depths of 20–50 cm^21^ or 40–80 cm^5^. However, this phenomenon was not noted in the oasis cropland of the present study, likely because the frequent and excess irrigation during the growing season greatly affected the root density and penetration.

Although maize was shallow rooted and mainly took up soil water from the surface (0–10 cm) during the growing season, only 24.7 ± 5.5% of the irrigation water remained in this layer after irrigation (Fig. 5); note that this value reflects the first day after irrigation. A study conducted in the North China Plain reported that the redistribution of irrigation water lasted for 7 days after irrigation^6^. Thus, the proportion of irrigation water retained in shallow soil layers would be further decreased over time. Over longer time scales, approximately 39% of the irrigation and rainwater was lost to soil layers below 80 cm (Table 1). Taken together, these findings show that a large amount of irrigation water was lost to drainage below the root zone. A previous study reported that the groundwater level of this area started to rise in 2003 after previously dropping approximately 10–30 cm every year until the end of 2002^36^. It is important to emphasise that the high groundwater table increased not only the nutrient loss due to the deep percolation process but also the risk of soil salinisation^30^. Nevertheless, a large number of croplands in the lower reaches would be desolated due to a water shortage, which in turn might further aggravate the desertification of this area. Therefore, reducing the amount of irrigation water and developing water-saving irrigation systems (e.g., spray, drip and infiltrating irrigation), as well as allocating water resources reasonably, will be important strategies to achieving sustainable development of agriculture in this area.

## Methods

### Study site

The research site was a superstation of the Heihe Watershed Allied Telemetry Experimental Research (HiWATER)^41^ that was located in an artificial oasis cropland in the middle reaches of the Heihe River Basin in north-western China (38°51′ N, 100°22′ E, 1550 m) (Supplementary Fig. S1). The annual precipitation is 128.7 mm, and the mean air temperature is 7.4 °C (1961–2010) according to the local meteorological station. Bulk density of the soil was 1.31, 1.37 and 1.51 g cm^-3^ for 0–5, 5–10 and 10–80 cm soil stratum, respectively. Soil texture is divided into the following particle grades: 2.0–0.05 mm (35%), 0.05–0.002 mm (38%) and <0.002 mm (27%)^33^. The staple crop in this region is maize (*Zea mays* L.) for seed production, which is harvested once per year. Maize was sown on 20 April (DOY 111) and harvested on 22 September (DOY 266) in 2012.

Our sampling plot (30 m long and 50 m wide) was surrounded by one road (2 m wide and 40 cm high) and three ridges (each 40 cm wide and 20 cm high). Transparent polyethylene films (70 cm wide and 0.007 mm thick) were used for water conservation. First, the soil surface was levelled and mulched with plastic films at approximately 60 cm intervals. Then, the edges of the film were buried to a width of 5 cm to prevent the wind from shifting the films. Therefore, the initial film cover rate in the field was approximately 54%. The maize seeds were planted along both inner sides of the films with plant and row spacing of 20 cm × 45 cm. Therefore, the roots of maize distributed in both the plastic-mulched and non-mulched soil. Small holes were made in the film to allow the maize to grow unhindered when the sprouts emerged (Supplementary Fig. S2). Irrigation water was spread over the plot by gravity without “tail-water” released. During the growing season, the cropland was irrigated 4 times with the upstream water of the Heihe River. The irrigation dates were 6 June (DOY 158, 111.6 mm), 2 July (DOY 184, 141.9 mm), 28 July (DOY 210, 149.7 mm) and 25 August (DOY 238, 149.7 mm) 2012. Because the topography was fairly smooth, irrigation water in the plot was mixed well. The irrigation amounts were calculated by the dataset of measurements on channel flow, which is available at the Data Center for Cold and Arid Region Sciences (http://westdc.westgis.ac.cn). The maximum leaf area index (LAI) was 4.4 m^2^ m^-2^, and the canopy height was 2.1 m. Further details about the site were provided in Huang and Wen^42^.

Auxiliary measurement instruments included an eddy covariance (EC) system (LI-7500, Licor Inc.; CSAT-3, Campbell Scientific Inc.; CR5000, Campbell Scientific Inc.)^43^,^44^,^45^ and a suite of micrometeorological sensors for measuring air temperature (HMP45AC, Vaisala Inc.), soil water content (ECH2O-5, Decagon Inc.) and precipitation (TE525MM, Campbell Scientific Inc.).

### Sampling and isotope analyses

One maize plant was randomly selected every 2–3 days per week (at midday), and the root crown was sampled to represent the *δ*^18^ O and *δ*D of the xylem water^46^. At the same time, two soil cores of 0–40 cm (divided into 0–5 cm, 5–10 cm, 10–20 cm, 20–30 cm and 30–40 cm depths) were sampled with a hand auger from both the plastic-mulched and non-mulched soil. Similarly, two soil cores of 0–80 cm were sampled weekly. The 0–40 cm soil cores were collected as described above, and the 40–80 cm cores were collected at every 10 cm depth (40–50 cm, 50–60 cm, 60–70 cm and 70–80 cm). After irrigation, the above samplings increased to once per day for 7 days. All of the samples were placed in vials and sealed with Parafilm immediately upon collection. During the study period, irrigation water and rainwater were collected. Samples for isotope analyses were kept frozen in a refrigerator (−15 °C to−20 °C) prior to water extraction.

Water in the root crown and soil was extracted with a cryogenic vacuum distillation system^47^. An extraction time of 60–90 minutes was required to obtain an un-fractionated water sample, which ensured an extraction percentage of water from the sample > 99.0%^9^. The isotopic composition of the liquid water samples was analysed using an Isotopic Ratio Infrared Spectroscopy (IRIS) system (Model DLT-100; Los Gatos Research, Mountain View, CA, USA) with a precision typically better than 0.1% for *δ*^18^O and 0.3% for *δ*D^48^. Because of the organic contaminants of water cryogenically extracted from plant tissues, the *δ*^18^O and *δ*D of xylem water measured by the LGR system needed to be corrected^49^,^50^. Schulz *et al*.^50^ reported that they were unable to create an ethanol correction curve for *δ*D. The slightly contaminated (BB < 1.2) xylem water *δ*D was corrected based on our calibration curves^49^. However, the correction curve for *δ*^18^O (y = − 0.15x + 0.99, *R*^2^ = 0.99, *p* < 0.001) was superior to that for *δ*D (y = − 0.23x + 1.09, *R*^2^ = 0.88, *p* = 0.04). Therefore, we only utilised the values of *δ*^18^O to identify the water uptake depth of maize and the infiltration of irrigation water in the subsequent analyses. During the study period, the average corrections were 1.6 ± 0.9% for *δ*^18^O and 2.9 ± 1.3% for *δ*D.

### Water source partitioning

The proportional contribution of the water sources for maize was evaluated using MixSIR^25^,^26^. MixSIR is a Bayesian stable isotope mixing model that incorporates uncertainty of multiple sources, isotope signatures and isotope fractionation (we can ignore it in this study, because there is no isotopic fractionations of oxygen isotopes during plant water uptake^51^). It allows users to input data and specify each of the mixture isotope signatures (i.e., xylem *δ*^18^O in this study) and the average and standard deviation (SD) of source isotope signatures (i.e., soil *δ*^18^O in this study) instead of the only arithmetic average of all the replicates. Although the widely used IsoSource model^23^,^24^ can also deal with the uncertainty associated with multiple sources, it does not formally incorporate the variations in isotope signatures.

To assess the influences of plastic films to water source predictions, we analysed the water uptake fractions from the different soil layers under various combinations of ratios (6:4, 5:5, 4:6, 3:7 and 2:8). To consider the influences of the spatial heterogeneity of the isotope data, we analysed the source contributions under different assumed (because no repeated samplings of xylem and soil water were taken in our study) SDs of the isotope data (0%, 0.6%, 0.9% and 1.5%). We also analysed these same data with the IsoSource model (the fractional increment was set at 1%, and the tolerance was set at 0.1%). In our study, the model solutions for a single day were presented as the median and 5th and 95th percentile values^26^. The solutions for a long period were presented as the average and standard deviation of the median values.

### Infiltration of irrigation water

If we assume that the water storage in soil layer *i* after irrigation (*W*_*ai*_) is composed of water in the same layer before irrigation (*W*_*bi*_) and water infiltrated from the above layer (*F*_*i-1*_), the following equation can be obtained:


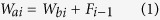


Based on the isotope mass balance, we obtain the following:


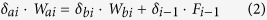


where *δ*_*ai*_, *δ*_*bi*_ and *δ*_*i*−1_ represent the *δ*^18^O of the above components. Rearranging the above equations:


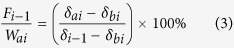


Therefore, the amount of irrigation water infiltrating to each soil layer can be quantified by the *δ*^18^O of different components and *W*_*ai*_. In addition, *δ*_*i−*1_ is *δ*^18^O of irrigation water (*δ*_*irri*_) when *i* = 1.

If we further assume that all of the irrigation water (*F*_irri_) infiltrates into the 0–80 cm soil layer, the *W*_*ai*_ of the 0–80 cm soil layer (*W*_*a*80_) can also be calculated:


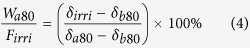


where *δ*_*a*80_ and *δ*_*b*80_ are the mean *δ*^18^O of the 0–80 cm soil water (weighted by soil water content) after and before irrigation, respectively. Then, we can compare *W*_*a*80_ with the direct measurement of the soil water storage (acquired by soil water content, m^3^ m^−3^) for validation.

We also calculated the deep percolation during the 4 irrigation events according to the soil water balance equation^7^,^30^:


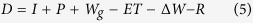


where *D* is the deep percolation below 80 cm (mm), *I* is the irrigation water (mm), *P* is the rainfall (mm), *W*_*g*_ is the capillary rise from groundwater (mm), *ET* is the evapotranspiration (mm) and *ΔW* is the change in soil water storage in the 0–80 cm soil layer (mm). *R* is the surface runoff (mm) and *W*_*g*_ is negligible during the growing season due to the lower water table (typically > 3.5 m). *R* is also negligible in this area because of the flat soil surface.

### Statistics

Statistical analyses were performed using the SPSS 17.0 program. The SWC, soil water *δ*^18^O and water source predictions were subjected to the One-way analysis of variance (ANOVA) to analyse the differences between plastic-mulched and non-mulched conditions (n = 62 for the 0–40 cm soil stratum, n = 17 for the 40–80 cm soil stratum) and between different soil combination ratios or different model types (n = 62) at α = 0.05. To detect the differences between *δ*^18^O in the 0–80 cm soil stratum (after combination), multiple comparisons were conducted using the least significant difference (LSD).

## Additional Information

**Accession codes:** The data for this paper are available at the Data Center for Cold and Arid Region Sciences: Data set 1: Data set of stable isotopic observation (doi: 10.3972/hiwater.108.2013.db); Data set 2: Data set of flux observation matrix (eddy covariance system of Daman Superstation Lower, doi: 10.3972/hiwater.096.2013.db): Data set 3: Data set of measurements of channel flow (doi: 10.3972/hiwater.123.2013.db).

**How to cite this article**: Yang, B. *et al*. Irrigation depth far exceeds water uptake depth in an oasis cropland in the middle reaches of Heihe River Basin. *Sci. Rep*. **5**, 15206; doi: 10.1038/srep15206 (2015).

## Supplementary Material

Supplementary Information

## Figures and Tables

**Table 1:** Deep percolation of irrigation water and precipitation after the 4 irrigation events.

Irrigationevents	*I*(mm)	*P*(mm)	*ET*(mm)	*ΔW* (mm)	*D* (mm)	*D*/(*I* + *P*)
I (DOY 158–184)	111.6	25.1	108.6	9.4	18.7	0.14
II (DOY 184–210)	141.9	36.9	103.1	−5.7	81.4	0.46
III (DOY 210–238)	149.7	26.8	105.2	−10.6	81.9	0.46
IV (DOY 238–265)	149.7	7.1	88.5	−4.7	73.0	0.47

**Table 2:** Sensitivity of the median water uptake fractions from different soil layers to the combination ratios of plastic mulched to non-mulched soil during the growing season.

Plastic mulch:Non-mulch	Range of mulch	Water uptake from		
		0–5 cm	5–10 cm	10–80 cm
6:4	+10%	+4.3 ± 5.8%	−1.8 ± 4.3%	−2.7 ± 3.5%
4:6	−10%	−6.6 ± 8.0%	+4.5 ± 8.9%	+2.1 ± 4.2%
3:7	−20%	−11.3 ± 9.2%	+6.0 ± 8.6%	+5.3 ± 5.6%
2:8	−30%	−15.2 ± 11.8%	+7.0 ± 10.0%	+8.3 ± 6.9%

For reference, the default ratio of plastic mulch to non-mulch is 5:5.

**Table 3:** Sensitivity of the median water uptake fractions from different soil layers to model uncertainty during the growing season.

Model types	SD of model	Water uptake from		
		0–5 cm	5–10 cm	10–80 cm
MixSIR_2	0.6%	−2.7 ± 3.7%	+2.2 ± 4.0%	+0.5 ± 3.5%
MixSIR_3	0.9%	−4.4 ± 6.8%	+3.6 ± 5.1%	+0.8 ± 6.9%
MixSIR_4	1.5%	−8.6 ± 11.3%	+4.6 ± 7.4%	+4.1 ± 6.8%
IsoSource	No	−0.5 ± 1.2%	+0.9 ± 2.4%	−0.4 ± 1.5%

For reference, the SD of default model (MixSIR_1) is 0.
