# Supplementary material for: Irrigation depth far exceeds water uptake depth in an oasis cropland in the middle reaches of Heihe River Basin
Source: Sci Rep. 2015 Oct 14;5:15206. doi: 10.1038/srep15206 (PMC4604461; doi:10.1038/srep15206)
Supplement: Supplementary Information [file srep15206-s1.pdf]

Supplementary Information to ‘Irrigation depth far exceeded water uptake depth of an oasis cropland in the middle reaches of Heihe River Basin’, by Bin Yang, Xuefa Wen and Xiaomin Sun.

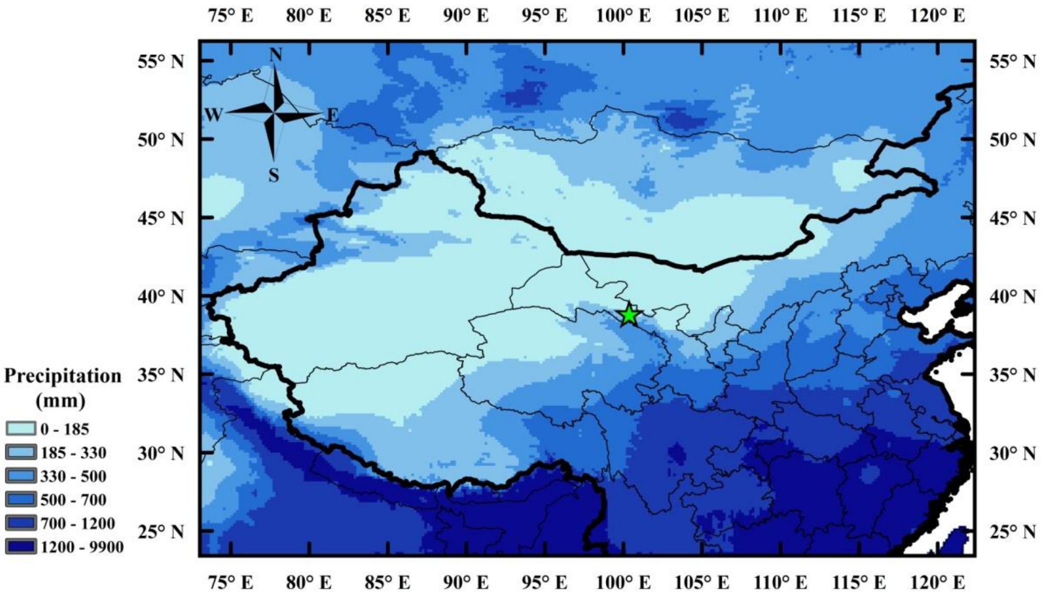

Supplementary Figure S1 | Spatial pattern of multi-year (1950-2000) mean precipitation according to Global Climate Data (http://www.worldclim.org/). The location of the study site is given by a star. The map was generated by ArcGis 10.0 software.

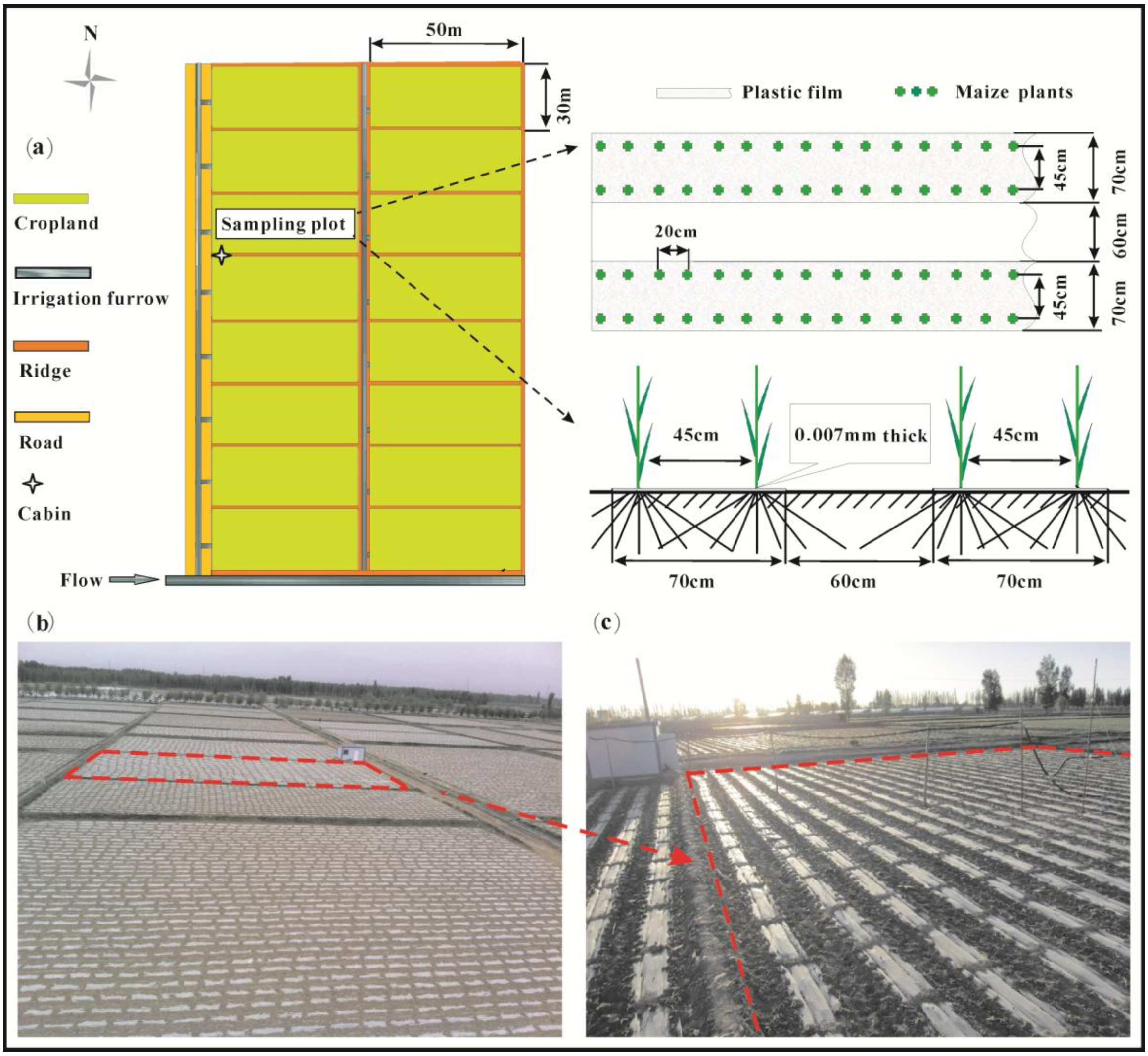

Supplementary Figure S2 | Schematic diagram (a) and photos (b and c) showing the sampling plot, irrigation furrow, mulched and non-mulched soil and the maize plants. The schematic diagram was generated by Corel DRAW 12.0 software.
